# Quantitative High-Throughput Screen Identifies Inhibitors of the *Schistosoma mansoni* Redox Cascade

**DOI:** 10.1371/journal.pntd.0000127

**Published:** 2008-01-02

**Authors:** Anton Simeonov, Ajit Jadhav, Ahmed A. Sayed, Yuhong Wang, Michael E. Nelson, Craig J. Thomas, James Inglese, David L. Williams, Christopher P. Austin

**Affiliations:** 1 NIH Chemical Genomics Center, National Human Genome Research Institute, National Institutes of Health, Bethesda, Maryland, United States of America; 2 Department of Biological Sciences, Illinois State University, Normal, Illinois, United States of America; Queensland Institute of Medical Research, Australia

## Abstract

Schistosomiasis is a tropical disease associated with high morbidity and mortality, currently affecting over 200 million people worldwide. Praziquantel is the only drug used to treat the disease, and with its increased use the probability of developing drug resistance has grown significantly. The *Schistosoma* parasites can survive for up to decades in the human host due in part to a unique set of antioxidant enzymes that continuously degrade the reactive oxygen species produced by the host's innate immune response. Two principal components of this defense system have been recently identified in *S. mansoni* as thioredoxin/glutathione reductase (TGR) and peroxiredoxin (Prx) and as such these enzymes present attractive new targets for anti-schistosomiasis drug development. Inhibition of TGR/Prx activity was screened in a dual-enzyme format with reducing equivalents being transferred from NADPH to glutathione via a TGR-catalyzed reaction and then to hydrogen peroxide via a Prx-catalyzed step. A fully automated quantitative high-throughput (qHTS) experiment was performed against a collection of 71,028 compounds tested as 7- to 15-point concentration series at 5 µL reaction volume in 1536-well plate format. In order to generate a robust data set and to minimize the effect of compound autofluorescence, apparent reaction rates derived from a kinetic read were utilized instead of end-point measurements. Actives identified from the screen, along with previously untested analogues, were subjected to confirmatory experiments using the screening assay and subsequently against the individual targets in secondary assays. Several novel active series were identified which inhibited TGR at a range of potencies, with IC_50_s ranging from micromolar to the assay response limit (∼25 nM). This is, to our knowledge, the first report of a large-scale HTS to identify lead compounds for a helminthic disease, and provides a paradigm that can be used to jump-start development of novel therapeutics for other neglected tropical diseases.

## Author Summary

Schistosomiasis, also known as bilharzia, is a tropical disease associated with high morbidity and mortality, currently affecting over 200 million people worldwide. Praziquantel is the only drug used to treat the disease, and with its increased use the probability of developing resistance has grown significantly. The *Schistosoma* parasites can survive for up to decades in the human host due in part to a unique set of antioxidant enzymes that continuously degrade the reactive oxygen species produced by the host's innate immune response. Two principal components of this defense system, thioredoxin/glutathione reductase (TGR) and peroxiredoxin (Prx2), have been recently identified and validated as targets for anti-schistosomiasis drug development. In search of inhibitors of this critical redox cascade, we optimized and performed a highly miniaturized automated screen of 71,028 compounds arrayed as 7- to 15-point dilution sets. We identified novel structural series of TGR inhibitors, several of which are highly potent and should serve both as mechanistic tools for probing redox pathways in *S. mansoni* and as starting points for developing much-needed new treatments for schistosomiasis. The paradigm presented here effectively bridges the gap between academic target identification and the first steps of drug development, and should be applicable to a variety of other important neglected diseases.

## Introduction

Schistosomiasis, also known as bilharzia, a debilitating disease resulting from the infection by the trematode parasite *Schistosoma* ssp. (*S. mansoni, S. mekongi, S. japonicum, S. haematobium,* and *S. intercalatum*) currently affects over 200 million people worldwide, mostly in developing countries [1]. A large percentage of those infected exhibit severe morbidity manifested as growth stunting, lassitude, and cognitive impairment [2], and an estimated 280,000 people die annually from the disease in sub-Saharan Africa alone [3]. The primary route of infection is via unsafe river and lake water, which is widely used in sub-Saharan Africa and Southeast Asia, among other regions, for irrigation, drinking, cooking, and bathing. Larval parasite forms (residing in and released by snails) can penetrate the skin of people contacting the water. The lifecycle of *Schistosoma* is exceedingly complex, with the parasite going through a number of stages both outside and inside the human host. Once inside humans, it can survive for years, even decades [4].

The need to control schistosomiasis is acute and efforts have been ongoing for years on three main fronts: prevention (via establishment and maintenance of sources of safe potable water), development of a vaccine, and use of drugs to treat the infection [1]. Although the number of schistosomiasis cases worldwide is indeed stunning, the number of drugs available to treat the disease is surprisingly small. Earlier in the 20^th^ century, schistosomiasis was treated with highly toxic antimonial compounds, of which the most common was potassium antimonyl tartrate (PAT, tartar emetic). During the past three decades the only drug used against the infection is praziquantel, which is administered orally, is stable, effective against all major schistosome species in a single dose, and relatively inexpensive [5],[6]. However, because of high reinfection rates, praziquantel must be administered on an annual or semi-annual basis. While its exact mechanism of action is unclear, praziquantel is thought to affect the parasites by disrupting calcium homeostasis [7],[8]. Preliminary reports of praziquantel-resistant cases, and the generation of praziquantel-resistant parasites in the laboratory [9]–[11] highlight the need for new drugs to treat the disease. Artemisinin has shown promise as a new drug for schistosomiasis [12] although its use for schistosomiasis may be restricted in areas of malaria transmission so that its use as an antimalarial is not put at risk. Simplified derivatives of artemisinin, the 1,2,4-trioxolanes, show promise and potential selectivity, but these, like the parent compound, are significantly less active against adult schistosome parasites [13]. Oxamniquine, used extensively in Brazil in the past, is effective only against *S. mansoni* and resistance has been reported further reducing its potential value in schistosomiasis control [10].

**Figure 1:**
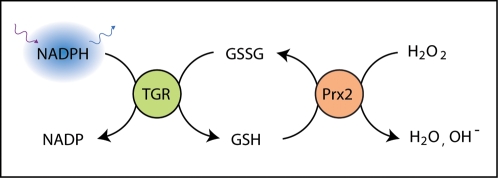
TGR/Prx2 assay principle.

Studies of the schistosome life cycle have focused on the fact it can survive for decades in the blood stream of the human host without being severely affected by the immune system and the associated assault by various reactive oxygen species (ROS). Since schistosomes do not have catalase to degrade hydrogen peroxide [14], other mechanisms must exist within the parasite to degrade ROS. Two uniquely positioned *S. mansoni* enzymes have been recently described that seem to act in concert to provide an effective antioxidant “firewall”. Thioredoxin glutathione reductase (TGR) is a multifunctional selenocysteine-containing enzyme that catalyzes the interconversion between reduced and oxidized forms of *both* glutathione (GSH) and thioredoxin (Trx), which are major contributors to the maintenance of redox balance in eukaryotes [15]. In contrast, humans possess two distinct enzymes, glutathione reductase (GR) and thioredoxin reductase (TrxR), which specifically recognize GSH and Trx as substrates, respectively [16]. The apparent replacement of two human enzymes by one dual-specificity worm enzyme has created a metabolic and regulatory bottleneck in which the inactivation of a single target, TGR, might have an enhanced deleterious effect on both the maintenance of parasite's redox balance and on its “antioxidant firewall”. Indeed, recent small molecule inhibition and RNA interference experiments have shown that inactivation of TGR has profound effects on *S. mansoni* survival rates both in culture and in infected mice [17]. Another component of the *S. mansoni* “firewall” are the peroxiredoxins (Prx), which are responsible for catalyzing the electron transfer to the main ROS agent hydrogen peroxide and, uniquely for schistosomes, from both GSH and Trx [18]. Thus, when TGR and Prx operate in concert, NADPH reducing equivalents are essentially transferred via TGR-catalyzed reaction to the oxidized forms of either Trx or GSH, while Trx or GSH in turn transfer reducing equivalents to hydrogen peroxide via Prx-catalyzed reactions (Figure 1).

Such improved understanding of the organisms responsible for neglected tropical diseases (NTDs) presents opportunities for new drug development. However, private-sector biopharmaceutical interest in NTDs has traditionally been limited due to high risk and low expected return-on-investment of these projects, though this is beginning to change with the advent of increased philanthropic and public-private-government partnership funding [19]. A significant problem that remains, however, is the significant gap in technologies, expertise, and cultures between academic and biopharmaceutical organizations [20]. At the US National Institutes of Health (NIH), the NIH Roadmap Molecular Libraries Initiative (MLI) was started in 2004 in part to address this problem. The MLI provides academic investigators with the pharma-scale infrastructure and technologies necessary to discover both chemical probes of physiology, and starting points for development of novel therapeutics for the rare and neglected diseases that are of less interest to the pharmaceutical sector [21].

The TGR/Prx work described here is the result of the first project officially accepted by the MLI in 2005. Since inhibiting either TGR or Prx can potentially lead to schistosome death [22], we chose to screen both enzymes in one assay as a reconstituted redox cascade. While TGR and Prx2 can be assayed individually, the separate assays are relatively less robust. TGR can be assayed in a relatively simple colorimetric assay by following the catalytic reduction of DTNB (5,5′ dithiobis(2-nitrobenzoic acid), Ellman's reagent) by NADPH; Prx2 at present can only be assayed with thioredoxin as a substrate together with TGR or thioredoxin reductase, or in a coupled reaction involving yeast glutathione reductase, and HTS-compatible assays [23] have yet to be developed [17],[18],[24]. By performing the high-throughput screen against both enzymes (present at equivalent levels in the assay), we were able to address both novel targets simultaneously while also combining target deconvolution and confirmation at the post-screen stage. In this report, we describe the miniaturization to 1536-well density of a cuvette-based assay for the TGR/Prx2 cascade which utilizes as a quantitative measure the decrease in fluorescence of the consumed NADPH substrate, the performance of a quantitative high-throughput screen (qHTS) [25] against 71,028 discrete compounds, and the initial characterization of several novel series of inhibitors. The application of qHTS, in which each library compound is assayed at a range of concentrations to generate a dose-response profile, facilitated triaging of actives for the purpose of structure-activity relationship (SAR) analysis and lead expansion.

## Materials and Methods

### Reagents

Nicotinamide adenine dinucleotide phosphate (NADPH), glutathione reduced form (GSH), hydrogen peroxide, Tween-20 and potassium antimonyl tartrate (PAT) were procured from Sigma-Aldrich. DMSO Certified ACS Grade was from Fisher. The screening assay was performed in 100 mM phosphate buffer pH 7.4 containing 10 mM EDTA and 0.01% Tween-20.

### Preparation of Recombinant *S. mansoni* TGR and Prx2

Recombinant TGR with a fused bacterial-type SECIS element was expressed in the *Escherichia coli* strain BL21(DE3) (Invitrogen) in the presence of pSUABC in LB medium supplemented with 20 µM flavin adenine dinucleotide following conditions for optimal selenoprotein expression as described [17]. TGR was purified to homogeneity on an adenosine 2′,5′-diphosphate agarose (Sigma) column equilibrated with TE buffer as described [17]. TGR concentration was determined from the flavin adenine dinucleotide absorption (ε_463_ = 11.3 mM^−1^cm^−1^). The pure protein was dialyzed against PBS and stored at −80°C.

Recombinant Prx2 in pRSETA was expressed in *E. coli* strain BLR(DE3)pLysS (Novagen) as described [18]. Briefly, after a 3-hr induction in 1 mM IPTG, cells were sonicated in 5% monothioglycerol (3-mercapto-1,2-propanediol) in 10 mM imidazole, 0.07 M Na_2_HPO_4_, 0.01 M NaH_2_PO_4_, and 0.15 M NaCl, pH 7.4. The supernatant was filtered and Prx2 was purified to homogeneity on a His Trap column (Amersham Biosciences). Protein purity was verified by SDS-PAGE. The purified protein was dialyzed against PBS and stored at −80°C until used.

### Compound library

The 71,028 member library comprised two main subsets: 59,692 compounds from the NIH Molecular Libraries Small Molecule Repository (www.mli.nih.gov), prepared as 10 mM stock solutions in 384-well plates and delivered by Biofocus DPI (South San Francisco, CA, http://mlsmr.glpg.com/MLSMR_HomePage/), and NCGC internal exploratory collection of approximately 11,336 compounds which consisted of several commercially available libraries of known bioactives (1280 compounds from Sigma-Aldrich (LOPAC1280 library), 1120 compounds from Prestwick Chemical Inc. (Washington, DC), 980 compounds from Tocris (Ellisville, Missouri), 280 purified natural products from TimTec (Newark, DE), 1980 compounds from the National Cancer Institute (the NCI Diversity Set)), 1408 National Institute of Environmental Health Sciences collection of known toxic compounds, as well as collections from other commercial and academic collaborators (three 1000-member combinatorial libraries from Pharmacopeia (Cranbury, NJ), 718 compounds from Boston University Center for Chemical Methodology and Library Development, 96-member peptide library from Prof. Sam Gelman's lab, University of Wisconsin, Madison, and 474 compounds from the University of Pittsburgh Center for Chemical Methodology and Library Development). The compound library (7 µL each in 1536-well Greiner polypropylene compound plate) was prepared as DMSO solutions at initial concentrations ranging between 2 and 10 mM. Plate-to-plate (vertical) dilutions and 384-to-1536 compressions were performed on Evolution P3 dispense system equipped with 384-tip pipetting head and two RapidStak units (Perkin-Elmer, Wellesley, MA). Additional details on the preparation of the compound library are provided in Inglese et al [25].

### Control plate

Titration of the known inhibitor PAT (PubChem CID6328158) was delivered via pin transfer from a separate plate to the lower half of column 2 of each assay plate. The starting concentration of the control, dissolved in 1∶1 DMSO∶water, was 1 mM, followed by five-fold dilution points in duplicate, for a total of eight concentrations.

### qHTS protocol

Three µL of reagents (100 µM NADPH in columns 3 and 4 as negative control and 100 µM NADPH, 42 nM TGR, 700 µM GSH, 83 nM Prx2 mixture in columns 1, 2, 5–48) were dispensed into 1536-well Greiner black assay plates. Compounds and control (23 nL) were transferred via Kalypsys PinTool equipped with 1536-pin array (10 nL slotted pins, V&P Scientific, Palo Alto, CA). The plate was incubated for 15 min at room temperature, and then a 1 µL aliquot of 400 µM NADPH/700 µM GSH was added, immediately followed by a 1 µL aliquot of 2.5 mM H_2_O_2_ to start the reaction. The plate was transferred to ViewLux high-throughput CCD imager (Perkin-Elmer, Wellesley, MA) where kinetic measurements (16 reads, one read every 30 sec) of the NADPH fluorescence decrease were acquired using 365 nm excitation/450 nm emission filter set. During dispense, the reagent bottles were kept submerged into 4°C recirculating chiller bath to minimize degradation. All screening operations were performed on a fully integrated robotic system (Kalypsys, San Diego, CA) containing one RX-130 and two RX-90 anthropomorphic robotic arms (Staubli, Duncan, SC). Library plates were screened starting from the lowest and proceeding to the highest concentration. Vehicle-only plates, with DMSO being pin-transferred to the entire column 5–48 compound area, were inserted uniformly at the rate of approximately one plate for every 50 library plates in order to monitor for and record any shifts in the background.

### Analysis of qHTS data

Time course data were collected on per-assay plate basis and were processed using in-house developed software. For each sample and at each individual concentration, 16 time points were processed using ordinary least squares regression to determine slope and intercept of linear fit. Additionally, a difference (delta) of last and first time point was generated for each time course. For activity calculations, delta values were chosen while the calculated slope, intercept, and the raw time-course data were stored in the database. Screening data were corrected and normalized and concentration–effect relationships derived by using the GeneData Screener software package (Basel, Switzerland). Percent activity was computed from the median values of the uninhibited, or neutral, control (48 wells located in column 1 and one-half of column 2) and the no-enzyme, or 100% inhibited, control (64 wells, entire columns 3 and 4), respectively. For assignment of plate concentrations and sample identifiers, ActivityBase (ID Business Solutions Ltd, Guildford, UK) was used for compound and plate registrations. An in-house database was used to track sample concentrations across plates. Correction factors were generated from the DMSO plate data and applied to each assay plate to correct for systematic errors in assay signal potentially resulting from issues with reagent dispensers or decrease in enzyme specific activity. A four parameter Hill equation [26] was fitted to the concentration-response data by minimizing the residual error between the modeled and observed responses. Outliers could be identified and masked by modeling the Hill equation and asking if the differences exceeded those expected from the noise in the assay.

The curve classification used is the same as described in Inglese et al. (2006) [25]. Briefly, concentration-response curves are placed into four classes: Class 1 contains complete concentration-response curves showing both upper and lower asymptotes and r^2^ values >0.9. Class 2 contain incomplete concentration-response curves lacking the lower asymptote and show r^2^ values >0.9. Class 3 curves are of the lowest confidence as they are defined by a single concentration point where the minimal acceptable activity is set at 3 SD of mean. Curves are classified as negative or positive depending on whether they exhibit signal decrease (apparent inhibition) or increase (apparent activation). Finally, Class 4 contains compounds that do not show any concentration response curves and are therefore classified as inactive.

A workflow was developed to facilitate a systematic approach in providing exhaustive analysis of structure activity relationships (SAR). First, a set of criteria used to define rules of determining an active set of compounds for the assay. These include decisions on inhibitors and activators, selectivity and counter screen information, curve class ranges, background fluorescence, etc. For this assay, compounds that showed signal activation (positive curve classes) were regarded as active due to fluorescence and were thus filtered out. The criteria are implemented as filters that are applied to rapidly define a core active set of compounds. This process eliminates many positive series that appear to have SAR and show reasonable titration response curves, but are not of biological relevance to the targets [23]. Next, the range of curve classes was limited to −1 through −3 to select for compounds showing signal decrease. Once an active set of compounds was identified, hierarchical agglomerative clustering with a 0.7 Tanimoto cutoff was performed using Leadscope (Leadscope Inc., Columbus, OH) fingerprints, which are ideally suited for two-dimensional scaffold-based based clustering [27]. For each cluster, maximal common substructures (MCS) were extracted, and a manual step of trimming the MCSs was performed to create a list of scaffolds. This clustering step typically has overlapping compounds and thus can lead to overlapping MCSs. This list of trimmed scaffolds is abridged to a canonical set. Each scaffold is then represented as a precise definition to indicate number of attachments, ring size variability, etc. All filters were then relaxed to include all negative assay data. In the initial clustering, a set of singletons was found. These compounds were reported upon separately with their individual activity profiles. SAR series and singletons were finally ranked by their activity profile.

### Follow-up testing of primary actives and analogs

Screening actives and analogues sourced as powders from the respective original suppliers (Sigma-Aldrich, NCI, Asinex, Chem Bridge, Tocris, Ambinter, and ChemDiv) were dissolved in DMSO to produce 10 mM initial stock solutions. The samples were then serially diluted row-wise in 384-well plate in twofold steps for a total of 24 concentrations, from 10 mM to 1.2 nM. Upon completion of the 24-point dilution, solutions from two 384-well plates were transferred to duplicate wells of 1536-well compound plate. The last two rows of the 1536-well plate did not contain any test compound and were reserved for placement of positive and negative controls. The assay protocol for confirmation was essentially the same as that described in the qHTS protocol section. A Flying Reagent Dispenser (FRD, Aurora Discovery, presently Beckman-Coulter) [28] was used to dispense reagents into the assay plates.

### Target-deconvolution assays

Assays were performed at 25°C in 0.1 M potassium phosphate, pH 7.4, 10 mM EDTA using 100 µM NADPH. The Prx2 assay was based on the reduction of H_2_O_2_ by Prx2 in the presence of GSH measured by the reduction of the GSSG produced in a coupled assay with yeast glutathione reductase monitored by observing the decrease in A_340_ nm due to consumption of NADPH (ε_340_ nm = 6.22 mM^−1^ cm^−1^) during the first three minutes [18]. The activity of TGR was determined with 3 mM 5,5′ dithiobis(2-nitrobenzoic acid) (DTNB, Ellman's reagent) [29] following the increase in A_410_ nm due to the production of 2-nitro-5-thiobenzoic acid (ε_412_ nm = 13.6 mM^−1^ cm^−1^) [17],[18].

## Results

### Assay Principle, Miniaturization, and Optimization

**Figure 2:**
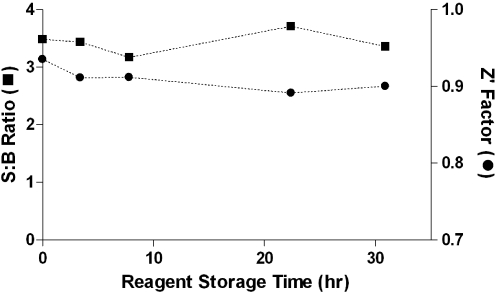
Screening reagents' stability as a function of storage time. Bottles containing buffer, enzyme, and substrate stock solutions, respectively, were prepared and kept at 4°C. At the selected time points, the bottles were connected to a liquid dispenser and the assay was performed as described in Methods. Signal-to-background ratio (S∶B) and Z′ factor were computed as the average of 32 wells.

The assay was initially developed and optimized using a spectrophotometer by following the decrease in absorbance at 340 nm associated with the consumption of NADPH substrate. During these studies, the main parameters of the assay, such as buffer conditions, concentration of each enzyme and substrate, DMSO and detergent tolerance, were tested and optimized (data not shown). The optimized assay utilized TGR and Prx2 at final concentrations of 25 nM and 50 nM, respectively. The substrates' final concentrations were 200 µM NADPH, 700 µM GSH, and 500 µM H_2_O_2_. The assay was miniaturized to 1536-well format by volume reduction and appropriate adjustment of stock concentrations of enzymes and substrates to reflect the volumes being combined. For example, the assay was started by the dispense of the two enzymes at 5/3 of their final concentration to account for the well volume increasing from 3 to 5 µL, while the hydrogen peroxide substrate was delivered as 5× solution to account for its dilution (1 µL to 5 µL final volume, see Materials and Methods, Table 1). All three types of reagents (enzymes, second aliquot of NADPH, and hydrogen peroxide) were tested and were shown to be stable overnight at 4°C, a requirement for the execution of an uninterrupted fully automated screen on the Kalypsys robotic system (Figure 2). In addition, the signal being monitored was changed from absorbance to fluorescence in order to: 1) improve the signal strength, as UV-shifted absorbance assays are generally difficult to scale to a 1536-well density (due to the combination of low extinction coefficient and short path length), and 2) minimize the quenching and inner-filter effects of a multitude of compounds which absorb light in this wavelength region [23].

**Table 1:** *S. mansoni* redox cascade qHTS protocol.

Step	Parameter	Value	Description
1	Reagent	3 µL	Enzyme and no-enzyme control solutions
2	Library Compounds	23 nL	57 µM to 2.9 nM titration series
3	Controls	23 nL	PAT titration
4	Incubation Time	15 min	Compound interaction with targets
5	Reagent	1 µL	Second equivalent of NADPH
6	Reagent	1 µL	H_2_O_2_ Substrate addition to initiate reaction
7	Assay Readout	365/450 nm	Fluorescence intensity kinetic read

Step Notes	
1	Black solid bottom plates, single-tip dispense, enzyme in columns 1, 2, 5–48, no-enzyme in columns 3 and 4.
2	Pintool transfer of library samples to columns 5–48 of the assay plate.
3	Pintool transfer of PAT titration to lower half of column 2.
4	Room temperature incubation in auxiliary hotel.
5	Solution of 400 µM NADPH and 700 µM GSH.
6	Hydrogen peroxide addition via 8-tip dispense.
7	ViewLux CCD imager, one read per 30 seconds for 8 min.

In a typical uninhibited reaction in 1536-well plate, the well fluorescence changed from 370 relative fluorescence units (RFU) to 180 RFU within an eight-minute window. This resulting outcome, if registered as an end-point reading, and assuming zero change in the background, would yield a signal-to-background (S∶B) ratio of only approximately 2.1. Therefore the assay was further improved by modification of the type of signal collected from single end-point to multipoint kinetic read. Thus, for each plate the reaction progress was recorded for 8 minutes at the rate of one fluorescence read every 30 seconds. Such kinetic mode data acquisition not only secured a more robust assay signal but minimized the interfering effects of dust and mildly fluorescent compounds (see Discussion).

### qHTS Performance

**Figure 3:**
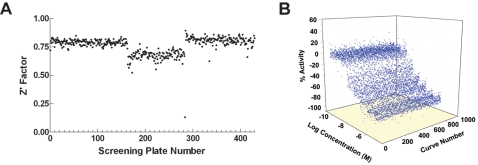
qHTS performance. Z′ trend (A) and intraplate control titrations (duplicate curves per plate) (B) as a function of plate number.

In total, 453 assay plates were screened in one uninterrupted robotic run lasting approximately 75 hours. Dispenser malfunction resulted in deteriorated signal in two plates and since the issue was noted in real time, the two 7-point libraries containing the problematic plates, plus two DMSO-only plates, were scheduled for re-screening immediately after the end of the main run. In this manner, the re-screened series were tested using the same batch of reagents as last series of the main screen. The assay performed robustly, yielding an average Z′ value of 0.76 [30]. Overall, the Z′ factor remained flat with the screen progression, with minor shifts tracking the introduction of new batches of the two enzymes (Figure 3A). The intraplate PAT control titration was stable throughout the screen progression, resulting in average IC_50_ of 14±8 nM and minimum significant ratio of 4.03 (Figure 3B) [31]. Each library compound was tested at a minimum of seven concentrations, ranging from 57 µM to 2.9 nM, and for each well, 16 time points were collected for a total of 9,562,432 data points. The screen and the preceding optimizations and validations consumed approximately 4.7 mg of TGR and 10 mg of Prx2. The overall materials cost of the screen (not including the cost of protein production) was approximately $5,200, or 0.85 cent per sample well, with approximately 80% of the costs associated with the assay microtiter plates and 15% attributed to NADPH.

### Analysis of qHTS data

Unlike traditional HTS, qHTS provides concentration responses for all the compounds screened and allows determination of an AC_50_ value, defined as the half-maximal activity concentration, for each compound in the primary screen. In qHTS concentration response curves are classified as belonging to one of four groups based on efficacy (response magnitude), presence of asymptotes, and goodness of fit of the curve to the data (r^2^). For the present screen, the activity associated with each well was computed from the change in fluorescence intensity over the time-course measurement period, normalized against control wells. In addition, the y-intercept of the reaction progress plot, typically equal to fluorescence at the first time point, was stored in the database and was used to further scrutinize purported actives. Compounds which showed activity but also had elevated y-intercept values were flagged as potential fluorescent artifacts.

### Overview of Actives

**Figure 4:**
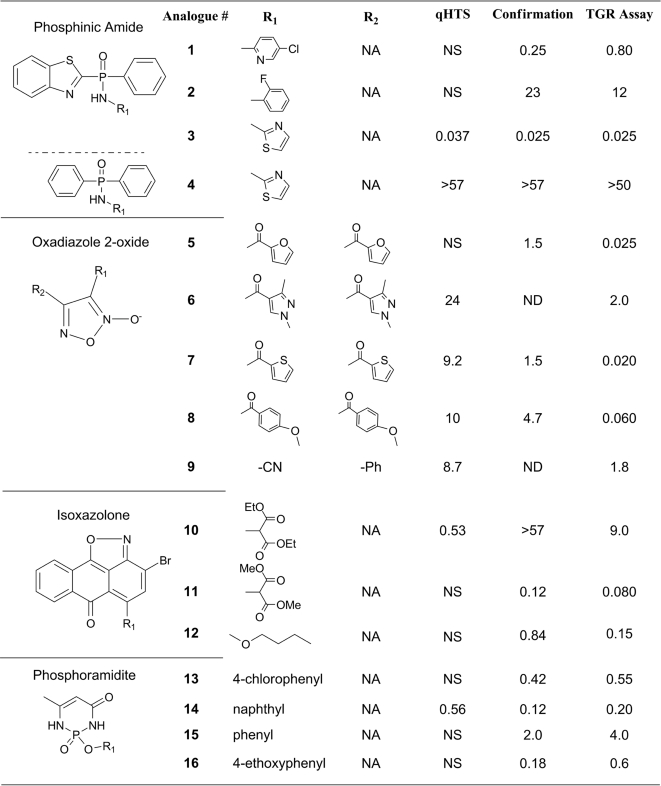
Structure and potency (IC_50_ in µM) of actives and series expansion analogues. ‘qHTS’ lists the IC_50_ values obtained from the initial high throughput screen of the collection; ‘confirmation’ represents the IC_50_ values obtained using the identical assay as in the qHTS from the compounds identified in qHTS and expansion series; ‘TGR Assay’ is the IC_50_ values obtained in an assay of TGR activity using DTNB as a substrate. NS, not screened (compound not in the screening collection), ND, not determined, NA, not applicable. IC_50_ values greater than 50 or 57 µM signify lack of fitted curve through the dose-response data, i.e. flat response within the range tested.

**Figure 5:**
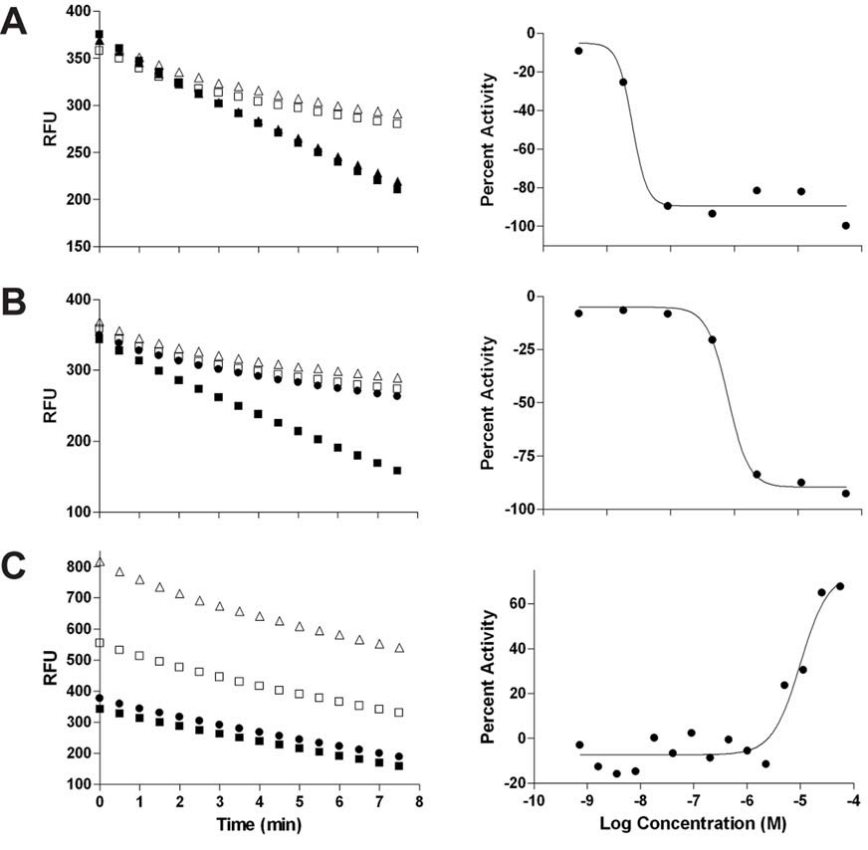
Example time-course plots derived from the primary screen, followed to the right by the corresponding concentration-response curves. (A, B) Non-fluorescent inhibitors, compounds 3 and 14; the decrease in fluorescence is inhibited at increasing compound concentrations while the starting fluorescence remains constant. (C) An example of an apparent activator (Ofloxacin): a highly fluorescent compound which appears to be reacting with one or more components of the assay. Four concentrations out of seven or fifteen are plotted in each of the time course plots for clarity; ▪, 3.2 nM; ▴, 16 nM; □, 10 µM; ▵, 50 µM.

Analysis of the qHTS results revealed 39 actives characterized by full concentration-response curves and IC_50_ values of better than 10 µM. After exclusion of antimony-containing compounds, as well as various mercury- and other heavy metal-containing molecules, the following series and singletons were selected for further studies after SAR analysis (Figure 4): oxadiazole 2-oxides (5 actives out of 29 analogues in collection, IC_50_ potency range between 8 µM and inactive), phosphinic amides (two compounds in the collection, one inactive and one active at 37 nM), phosphoramidite (singleton active, IC_50_ of 560 nM), and isoxazolone (singleton active, IC_50_ of 530 nM). In addition, a weaker series, quinolinyl sulfonamides (8 actives out of 47 total analogues in collection, IC_50_ potency range between 0.6 µM and inactive), was identified but noted to contain a number of both active and inactive members which were strongly fluorescent as judged by the extreme intercept values recorded during the screen. In contrast, neither the oxadiazole series nor the singleton actives exhibited any detectable autofluorescence (Figure 5).

In addition to inhibitors, the screen yielded a number of apparent activators, that is, compounds for which the increase in concentration led to a fluorescence intensity change *greater* than that of the neutral control. Upon examination of the time-course plots associated with these activators it became evident that the signal enhancement originated from high starting fluorescence which decreased during the observation window and in many cases entirely obscured the assay-driven NADPH fluorescence change (Figure 5C). While some of these compounds might be fluorescent substrates for either TGR or Prx2, which get converted to non-fluorescent products, a large number might simply be reactive towards any one or more components of the assay milieu (GSH, NADPH, and/or H_2_O_2_). As such, their confirmation and mode of action is subject of separate study.

### Series expansion and confirmation

In order to further confirm the qHTS actives and to expand the actives series, especially around the otherwise attractive singletons, powder samples were purchased from the original compound vendors and processed as described in the Methods. In addition to qHTS-identified compounds, untested analogues of the singletons were also procured in an attempt to support the singleton findings by the generation of small SAR series. The comparison of qHTS results, where applicable, and re-test results from independently acquired powder samples are shown in Figure 4, first and second data columns, respectively. The overall confirmation rate was excellent with the exception of the sulfonamide series of actives, which showed wide shifts between qHTS and confirmatory assay (results not shown). The apparent lack of confirmation for this series was consistent with the aberrant fluorescent values associated with many of its members. An analogue series built around a lower-potency benzoindolone singleton (NCGC00038549, IC_50_ of 3.9 µM) failed to yield activity against the screening assay and against both TGR and Prx2 individual assays. The original activity of that singleton was therefore deemed an artifact.

Gratifyingly, the previously-untested analogues of the phosphinic amides (compounds **1**–**4**), phosphoramidite (**13**–**16**), and isoxazolone (**10**–**12**) actives all showed activity with various degrees of potency, supporting and expanding the qHTS findings. Specifically, the “gap” in potency between the highly active **3** (NCGC00042730, qHTS IC_50_ of 37 nM, confirmed at 25 nM on re-test) and the inactive distant analogue **4** (NCGC00064648) was filled partially by the newly-acquired analogues **1** (NCGC00093512, IC_50_ of 247 nM) and **2** (NCGC00093512, IC_50_ of 23 µM). Similarly, increased potency was achieved by the addition of analogues to the phosphoramidite (from a singleton IC_50_ of 0.5 µM to a range of 0.2–2 µM) and the isoxazolone (from a singleton IC_50_ of 0.5 µM to a range of 0.1 µM to 9 µM).

### Target deconvolution

After establishing the activities of primary hits and new analogues against the screened dual-enzyme system, the compounds were further subjected to target deconvolution experiments. When tested against Prx2 in a GR-coupled hydrogen peroxide reduction assay none of the selected compounds showed activity up to the 50 µM top concentration tested (and by extension, none were active against GR, an enzyme related to TGR). Prx2 was therefore ruled out as the target of any of the actives identified in the screen. Results from the TGR assay are shown in the last column of Figure 4. The majority of active compounds demonstrated approximately the same, and in some instances improved (most notably with **7** and **8**), potency against the isolated TGR as they did against the dual-enzyme system. These results not only confirm the initial findings from the screen, but also further support the hypothesis of TGR being the sole target of these actives. Additionally, all members of the sulfonamide series were inactive against both Prx2 and TGR, further strengthening the argument that their initial classification as actives was due to fluorescence interference originating from either the compound, impurities, or product(s) of its breakdown.

## Discussion

The stability, relatively low cost, and effectiveness of praziquantel has practically created a dependency on this single drug to treat schistosomiasis. Both the success of praziquantel and the general lack of incentives for large pharmaceutical companies to embark on research and development in the area of tropical diseases have led to a fairly dry pipeline for both drugs to treat schistosomiasis and basic research tools to study the lifecycle of this important parasite. To this end, we implemented a highly-miniaturized automated screen of the NCGC small molecule collection in an attempt to identify novel inhibitors of *S. mansoni* TGR or Prx2, both of which have been recently validated as crucial *S. mansoni* enzymes and have been proposed as targets for drug development. Prior to HTS adoption, the assay employed monitoring NADPH absorbance. While such a format is very convenient, offering fast access to kinetic data via the use of standard spectrophotometers, measuring absorbance in the UV region in 1536-well density is rarely practical. A significant fraction of organic molecules, as well as dust and buffer components, absorb in the 350 nm range, thereby introducing unacceptably high levels of interference. Additionally, the relatively low extinction coefficient of NADPH coupled with the short optical path length of the plate well significantly reduces the signal available for detection. Because NADPH is naturally fluorescent, emitting at ∼450 nm, while its oxidized counterpart NADP is not, we switched the detection platform for the coupled reaction from absorbance to fluorescence, a step that parallels the application of profluorescent substrates in assays for phosphatases and proteases [23], with the main difference being the fluorescence change trending from high to low in this reaction.

Because of the anticipated fluorescence interference from compound library members in this blue-shifted detection region and because the output generated from NADPH is not very strong (due to the combination of low extinction coefficient and quantum yield), we further modified the detection format of the assay to measure the reaction progress in kinetic mode as opposed to collecting a single end-point read. While kinetic, or time-course, measurements are routinely performed during assay development in low-throughput settings, their practical implementation during automated large-collection screens is not trivial. Unless the reaction under study is slow, only a fast-scanning reader or whole-plate imager (such as the ViewLux) can allow positionally-unbiased and rapid repeated measurements of 1536-well plates without significantly slowing down the overall plate processing speed. The collection of at least a two-point time course allows the effects of dust and fluorescent but otherwise inert library members to be subtracted out to reveal the true reaction course. Because the first time-point values (when the enzymatic reaction has produced minimal amount of product) associated with each compound well are stored in the database, a further analysis can be performed to flag interfering fluorescent library members [32]. An added benefit is that the signal-to-background computed from kinetic measurements significantly improves relative to end-point data and thus allows screening under conditions of low substrate conversion [33]. While in this screen we collected a total of 16 points per well, further optimization of the assay conditions could have resulted in shortened read time without the loss of sensitivity.

The primary screen against the TGR/Prx2 cascade was performed in Quantitative High Throughput Screening (qHTS) format. In qHTS, every compound in the collection is tested over a range of concentrations, spanning from tens of micromolar to low nanomolar, to generate a complete concentration-response profile. As such, qHTS is best described as high throughput pharmacology, since as a result of its application, not only are potencies and efficacies assigned to each active compound but also false positives and negatives due to outliers associated with individual concentration responses are easily identified in the context of titration. Additionally, due to the built-in replicates in the testing of each compound, the need for laborious and infrastructure-intensive cherry-picking, original-result replication, and dose-response characterization are eliminated. In our present assay, the application of qHTS enabled us to not only skip the direct confirmation steps but also to combine the actives verification from independently-sourced powders with series expansion around limited SAR or singletons. In traditional single-concentration screening, singleton actives are necessarily treated with great caution given statistical uncertainties. In this study, the qHTS paradigm allowed us to confidently select the potent phosphinic amide, isoxazolone and phosphoramidite singletons **3**, **10** and **14** (Figure 4) for further testing and that selection was later validated by the excellent confirmation of those actives and the successful expansion of the series. Separate, but equally important, is the aspect of reliability and robustness of screening data. qHTS, with the combination of dose-survey and replicate points, indeed offers uniquely rich and robust data sets for deposition in recently established public databases, such as PubChem. Additionally, in order to minimize the interfering effect of promiscuous inhibitors acting via colloidal aggregate formation [34],[35], we included detergent in the assay buffer.

Throughout the entire screen, the assay performed in a robust manner, yielding an average Z′ value of 0.76. Overall, the Z′ factor remained flat with the screen progression, with minor shifts tracking the introduction of fresh batches of enzyme. The availability of periodically computed Z′, signal-to-background, and activity heatmaps throughout the screen progression, made possible by the development of fast data-processing tools in-house, significantly improved our response time when screen complications arose. For example, the dip in Z′, also accompanied by noisy activity heatmap (not shown), was noted almost in real-time and this allowed the appropriate concentration series to be scheduled for re-run within the same screening session.

Figure 3B presents a cumulative plot of all intra-plate concentration-response curves of PAT throughout the entire screen. The narrow range of observed IC_50_ values serves as a further indication that the screen performed robustly from a standpoint of enzyme activity and responsiveness to inhibition. The titration curve displayed stability throughout the screen despite the fact that PAT is only partially soluble in DMSO and required formulation in high-percent water, leading to concerns about evaporation-related variability. Analyzing the trend in the intra-plate control as a function of screen progression allows one to ascertain the ‘health’ of the screening system as a whole, because the variations or dramatic shifts in potency of the control could be due to not only a deterioration in enzyme quality (which could otherwise be detected from an shift in the S∶B value) but also to problems with the pintool delivery of compounds. The absence of abrupt and significant shifts in the intra-plate control curve allows us therefore to conclude that the compound transfer remained uniform throughout the screen.

The screen identified numerous arsenic, antimony, mercury, and other heavy-metal containing compounds (data not shown; for complete set of actives, see PubChem, AID 448). The antimony-containing compounds were largely similar to PAT and were therefore expected to be identified by this assay. Likewise, Hg-derivatives inhibited the enzymes strongly, as expected. While PAT and the gold-containing drug auranofin had been shown to inhibit thioredoxin reductase and TGR [17],[36],[37], and while more recently arsenic trioxide has shown anticancer activity and its effect has been ascribed to Trx reductase-mediated apoptosis [38], we restricted our analysis to novel nonmetal-containing compounds, primarily due to the fact that heavy metal-containing compounds frequently exhibit non-selective inhibition against a broad panel of enzymes and because nonmetal novel chemotypes against these previously-unscreened targets appeared to offer the greatest promise for optimizing potency and specificity. In this regard, the screen was successful, having resulted in identification of several distinct series of TGR inhibitors. It is noteworthy that the top actives from some series, such as the phosphinic amide **3**, oxadiazole 2-oxide **7**, and isoxazolone **11**, yielded IC_50_ values close to the final TGR assay concentration (25 nM in qHTS and confirmation and 15 nM in the TGR individual assay), thus approaching the limit of the detectable potency range. In terms of the lead actives there are several interesting points. For instance, the role of the benzothiazole heterocycle within the phosphinic amide series is apparently critical for inhibition as illustrated by the comparative values of analogues **3**, active and containing a benzothiazole moiety, and **4**, inactive and devoid of benzothiazole (Figure 4). The oxadiazole series contains several symmetric heterocyles (a function of their synthetic ease) and are known NO donors [39],[40]. The presence of two phosphorus based small molecules may well relate to the presence of a selenocysteine in TGR and the relative electrophilic nature of this functionality. Studies to expand upon selected lead actives and further understand their mechanism of action are currently underway. Furthermore, in the Prx2 deconvolution assay, none of the top actives were found to inhibit GR, an enzyme closely related to TGR. This suggests that selective activity against the parasite (which lacks GR) and less toxicity to humans (who have GR) can be achieved.

In summary, a kinetic-based qHTS against a pair of novel, validated targets from *S. mansoni* allowed fast and reliable identification of compounds active against this critical redox cascade. We have identified several novel structural series of TGR inhibitors, several of which are highly potent and should serve both as mechanistic tools for probing the redox balance in *S. mansoni,* and starting points for developing medicinal leads for much-needed new treatments for schistosomiasis. The work presented here effectively bridged the gap between academic target identification and the first steps of drug development for an important neglected disease [20]. Generalization of this paradigm to other neglected diseases could prove be a powerful approach to catalyzing new therapeutic development for NTDs.
